# Why twenty amino acid residue types suffice(d) to support all living systems

**DOI:** 10.1371/journal.pone.0204883

**Published:** 2018-10-15

**Authors:** Robert P. Bywater

**Affiliations:** Francis Crick Institute, London, England; CPERI, GREECE

## Abstract

It is well known that proteins are built up from an alphabet of 20 different amino acid types. These suffice to enable the protein to fold into its operative form relevant to its required functional roles. For carrying out these allotted functions, there may in some cases be a need for post-translational modifications and it has been established that an additional three types of amino acid have at some point been recruited into this process. But it still remains the case that the 20 residue types referred to are the major building blocks in all terrestrial proteins, and probably "universally". Given this fact, it is surprising that no satisfactory answer has been given to the two questions: "why 20?" and "why just these 20?". Furthermore, a suggestion is made as to how these 20 map to the codon repertoire which in principle has the capacity to cater for 64 different residue types. Attempts are made in this paper to answer these questions by employing a combination of quantum chemical and chemoinformatic tools which are applied to the standard 20 amino acid types as well as 3 “non-standard” types found in nature, a set of fictitious but feasible analog structures designed to test the need for greater coverage of function space and the collection of candidate alternative structures found either on meteorites or in experiments designed to reconstruct pre-life scenarios.

## Introduction

The search for organic compounds that could act as components or precursors to key prebiotic chemicals has been pursued actively for more than 60 years [1–3]. Only a relatively small number of such candidates numbering 50 or so have been found. This number is vanishingly small when compared with the estimated potential population of chemical space (“space” here used in its generic, mathematical sense). Chemical space has been defined [4]as the ensemble of all possible molecules, which is reckoned to contain at least 10^60^ organic molecules below 500 Da. It may be that millions of compounds have yet to be discovered in outer space, but the number of actual known compounds is tiny compared to this number. Meanwhile on Earth both biology and the ingenuity of mankind are capable of generating millions of different chemical structures within tiny enclosed spaces. The contrast between the chemical sterility of the known outer space and Earth could not be greater. Of course, biological evolution has had a lot to do with the production of these millions of compounds (still nowhere near the estimate of Reymond et al. however), but the chemical evolution that led up to the emergence of life had to make do with the 50 discussed here, of which 19 are the extant and still necessary L-amino acids together with the achiral glycine and at some stage an additional 3 types.

The extent to which chemical space has been populated by prebiotic chemicals has been considered before and advanced chemoinformatic tools have been employed in these searches [5–9]. In particular, it was pointed out [6]that “… data of amino acid frequencies formed by these diverse mechanisms and … regardless of the source, these 10 early amino acids can be ranked in order (can be predicted by thermodynamics) of decreasing abundance in prebiotic contexts .”. It is in this spirit (chemoinformatics and thermodynamic constraints) that this search is continued here, using several new and some older methodologies.

Proteins exert a vast repertoire of functions that are critical for the survival of all living organisms. These include structural, catalytic, regulatory functions, transport of bioactive materials, signal transduction and gene transcription roles and many others. This huge diversity of function is all the more remarkable considering that the chemical make-up of proteins is seemingly deceptively simple–an alphabet of only 20 amino acid residue types provides the platform or stage upon which all of these complex roles can be played. These 20 residue types possess, through the chemical nature of their side-chains, almost all of the required chemical functionality. One needs to say “almost all” because in certain cases, there is considerable extra support provided by post-translational modifications, addition of cofactors or metal ions *etc*. but the amino acids themselves supply the required information for the correct attachment of these auxiliaries. Mention has already been made of three additional, relatively rare, proteinogenic amino acid types, two of which are encoded by overriding a stop codon, UAG in the case or pyrrolysine and UGA in the case of selenocysteine [10–14]. The major focus in this work is the 20 standard or canonical amino acid types, but although these two additional types have so far not been found outside of the biosphere, they play an important role in “post-RNA” biology so they are included in the analysis which follows, along with a third newcomer, selenomethionine [15] other candidate amino acid analogs as specified below.

The selection of the 20 standard residue types was made early on in evolution–their appearance predates RNA and DNA and it is highly likely that they already played a vital role throughout prebiotic chemical evolution (~4 Gyrs ago) [7,8]. The questions that arise now and even earlier [16] are: why did these 20 “canonical” amino acid types come to populate the protein world, and why were others (in principle, many possible alternatives [16]) not selected?

These questions are relevant to the biochemistry of today as well as for considerations of their role in the origin of life (OoL). We have recently been informed about the nature of the organic chemical payload aboard comet 67P [17]but these were mainly very primitive structures including many nitrogen bearing species but no sulfur (or selenium) containing species, and four organic compounds methylisocyanate, acetone, propionaldehyde and acetamide, but no amino acids. In time we will get information about a number of planets within the solar system as well as innumerable exoplanets. Although there may be some surprises, it would seem that, based on current data, “universally” is a valid adverb to use. If this is the case, the answer must require us to find out what are the factors pertaining to proteins that restrict the uptake of whatever monomers that organic chemistry can offer under what are, at the outset anyway, harsh conditions.

Finally, a most pertinent question mentioned in the **Abstract** and already present on an earlier list [16]of questions: why only 20 and not 64 amino acid types. This question is addressed in this paper.

## Methods

Key to this paper is an understanding the notion of and value in using the various definitions of molecular complexity. If one is ever to understand how life could emerge from a prebiotic chemical library of 50 compounds (among the vast array of 10^60^ possible chemical candidates most of which we will never encounter) it is necessary to resort to quantitative measurements that reflect the likelihood of any given compound ever being made. There are two (complementary) approaches to this, one based on mathematical descriptors of molecular structure (chemoinformatics) and the other based on quantum chemistry. Both approaches are applied here. Note that in the present work, molecular complexity based on structure alone is used, earlier usage of “synthesizability” metrics [7,8]is not relevant in this context since we are interested in spontaneous production of the various molecular species, not their synthesis involving elaborate procedures and cycles of purification at the lab bench.

In order to investigate these questions a panel of 58 compounds including the 20 canonical amino acids was subjected to a rigorous statistical audit of their structural and quantum chemical properties. The choice of candidate compounds for this study was dictated by known facts concerning the appearance of amino acids, their precursors and analogues in the Murchison meteorite [18]and in the classical Miller experiment [1–3]. The panel of compounds selected here are those listed in **S1–S5 Tables** and identified by name in **S6 Table**. In the work described herein these sets are referred to as *mrchsn* for Murchisonand *mllr* for Miller and Miller-Urey respectively. (For compounds numbered 1–30 see: http://en.wikipedia.org/wiki/Non-proteinogenic_aminos_acids). It should be mentioned that many experiments have been carried out since the original Miller experiments under different conditions, but to all intents and purposes, the same chemicals are produced [5,6,19–21]. The chemicals considered in this work were initially divided into two groups, L-amino-acids that are members of the “canonical set” (12 of the 20) (**S2 Table**) and other amino-acid-like compounds (**S1 Table**) referred to as “candidates”. It is important to stress that most of these candidates are not amino acids. They cannot be incorporated into peptides, since they lack one or other or both of the defining chemical functional groups for forming a peptide bond. There are some exceptions to this which are discussed below.

Further, since we wish to investigate chemical evolution, it is most important to consider what chemicals (relevant to modern biochemistry) were not present in these two sources. Consequently, a third group (**S3 Table**) was added: these were the remaining 8 of the set of 20 which have not been detected in meteorites or in OoL simulation experiments. Of course, these 8 as well as all of the other peptidogenic amino acid types are, in the post-RNA era, produced by enzyme catalysis, but they were present prior to the appearance of RNA and proteins. All 20 are to be regarded as prebiotic, and the 8 not found in simulation experiments or on meteorites would owe their origin to terrestrial catalysts as discussed below. The established (from meteorite evidence and Miller experiments) 12 amino acids conform to the same basic 12 structures that we have today, There could have been many elaborations of these structures but they have not been witnessed. The reason for this is that all such elaborations such as chain-branching or introduction of new chiral centers (which usually follows from chain-branching) comes at a cost which is measurable in terms of complexity and synthesizability and observable in terms of occurrence. These stringent requirements also operate to confine the next set to the 8 we still observe today. Apart from isoleucine and threonine there are no amino acid types with more than one chiral center with the sole excepton of pyrrolysine (compound 51 in **S4 Table**) which has two extra chiral centers. This is reflected in the very high complexity score for this amino acid as described later.

The protein world is (apart from some prosthetic groups and posttranslational modifications) mostly populated by atom types H, C, N and O but we have already mentioned amino acids containing Se and they are included here (compounds 52–54 in **S4 Table**).

There could be, or could have been, other ways to elaborate amino acid structures by exploring aromaticity space or making alternatives to existing functional groups such as -CO2 (-) and -NH3 (+). To investigate this a small set of amino acids was designed in a determined effort to study an area of “function space” previously unexplored either by chemical evolution or even synthetically. The members of this set were chosen carefully with a view to exploring function space with good coverage but in a nonredundant way. This took the form of extending aromaticity space by replacing the single benzene ring of phenylalanine with a naphthalene moiety (2-amino-3-naphthylpropanoic acid, compound 55 in **S5 Table**) and replacing the imidazole moiety of histidine with triazoles (2-Amino-3-(1H-1,2,3,triazol-4-yl)propanoic acid, compound 56 and 2-Amino-3-(1H-1,3,5,triazol-4-yl)propanoic acid, compound 57) and tetrazole (1H-1,2,3,5,tetrazol-4-yl)propanoic acid, compound 58). The imidazole sidechain of histidine ionizes within the range of pH of biological systems which gives it its catalytic properties, while triazoles are always positively charged and tetrazoles negatively charged at physiological pH. These analogs are not found naturally and indeed would be quite tricky to synthesize.

It was not considered necessary to further explore aliphaticity space (compounds with high logP as discussed below), since this has been adequately covered by early chemical evolution (many compounds in the first group, in particular no. 14). In any case, extending aliphaticity by studying higher homologues or very branched sidechains would rapidly encounter two disqualifications, that of low solubility and, from the structural point of view excessive redundancy without any compensating gains in terms of potential for enhanced molecular recognition or function. Likewise, increasing charge by adding extra charged groups would be self-defeating as soluble counterions will soon mop up the extra charges while there would be a concomitant increase in “complexity cost” without any gain in functionality.

The five compound groups are discussed in detail below.

**Fig 1:**
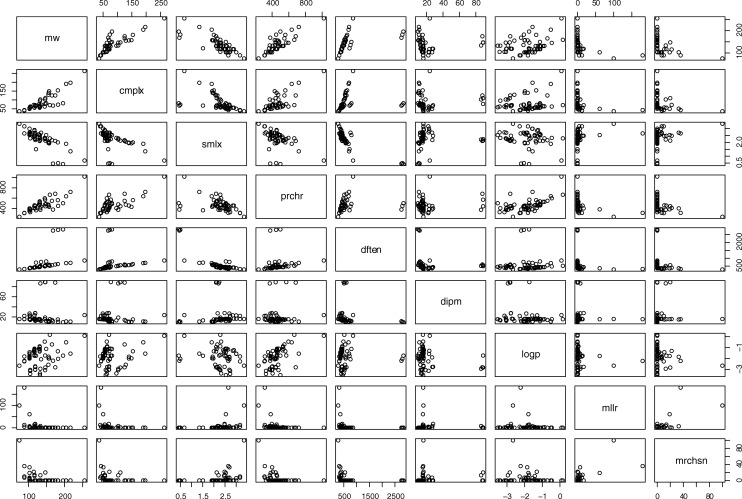
Pairwise correlations between variables used in the R program.

The input models of the selected compounds were constructed using the YASARA modeling package [22].They were first optimised by semiempirical quantum chemical calculations under the PM3 Hamiltonian and stored in PDB, XYZ and SMILES formats. The XYZ files were used as input in order to obtain accurate electronic structural data using *ab initio* quantum methods of the B3LYP/6-31G* type implemented within the MOLCAS program [23]. This was chosen because the compounds are all closed-shell structures with no excited states or radicals involved. Calculations of the latter type need to be set up with care and attention to many factors other than structure and special basis sets need to be chosen. But for compounds like the ones studied here B3LYP is perfectly adequate. An extra complication in the case of the seleno- compounds (Nos.: 52, 52 and 54) is that a relativistic correction (Basis: ANO-RCC-VDZP) was required to provide an accurate scf convergence, which increased the computational overheads somewhat. Since there is no specific information about the nature of the environment that applies to all cases (it could be vacuum, water, mineral surfaces, lipid bilayer depending on the case) the dielectric constant was set to unity. (It has been pointed out [20]that in all of the Miller experiments and subsequent attempts to repeat them under different conditions, no account was made of geochemical conditions). From the B3LYP output data, DFT energies (*dften*) and dipole moments (*dipm*) for each compound were extracted. These variables summarise different electronic attributes of the molecules: *dipm* is the net dipole moment for the molecule. Dipole moments are normally split along the three major moment of inertia axes of the molecule, but as there is no common way to align all the molecules (if there exists such an alignment, it is precisely the direction of this net dipole vector), it is admissible for this work to use the magnitude of this vector as a measure of polarity. The DFT energy of the molecule, *dften*, correlates strongly with complexity and molecular weight (see **Fig 1**), as one would expect.

**Fig 2:**
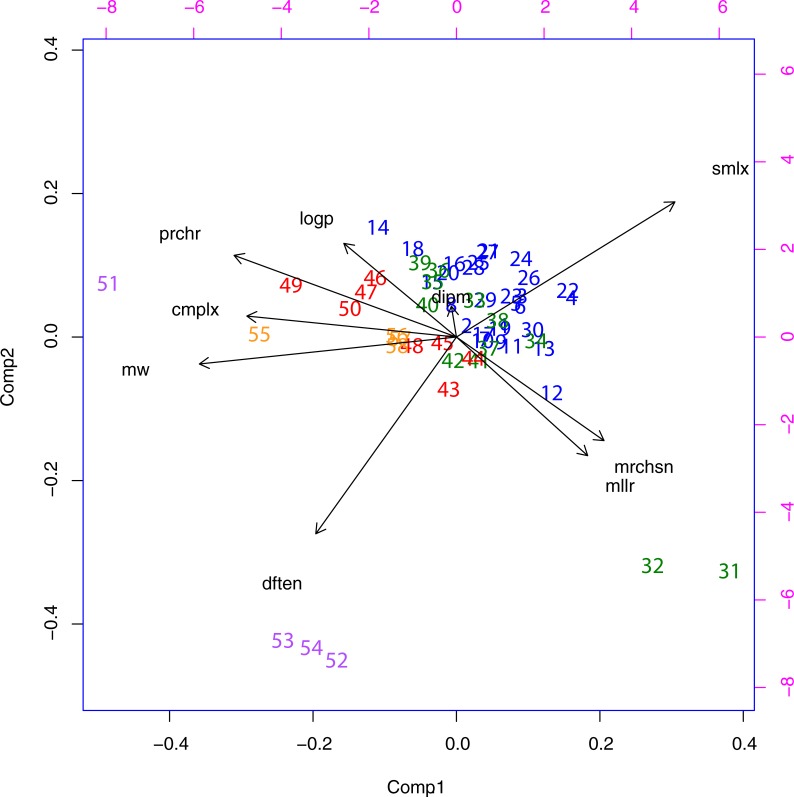
PCA analaysis of the entire data set (Groups 1,2,3, & 4).

**Fig 3:**
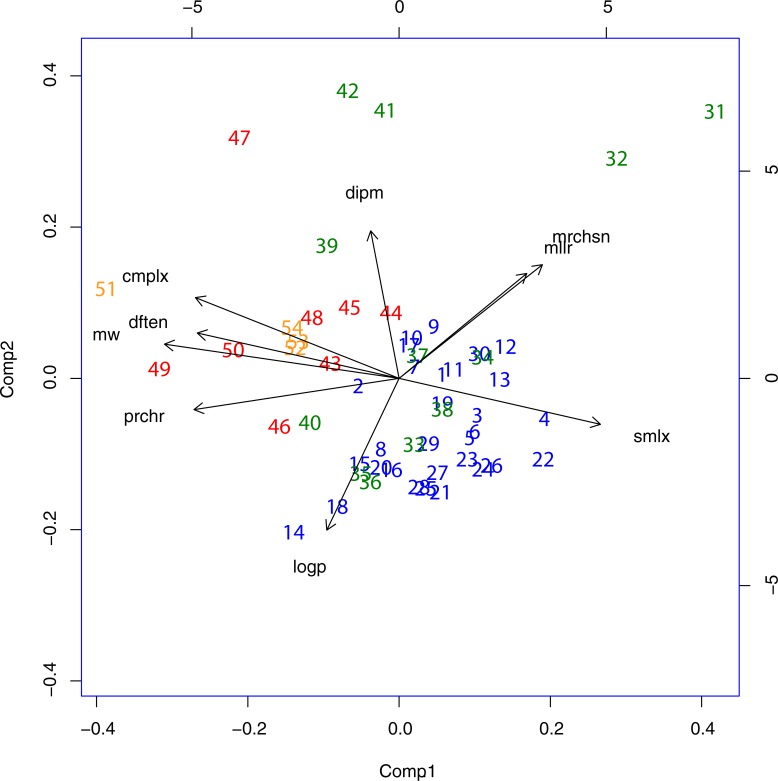
As in Fig 2 but with only Groups 1, 2 & 3.

**Fig 4:**
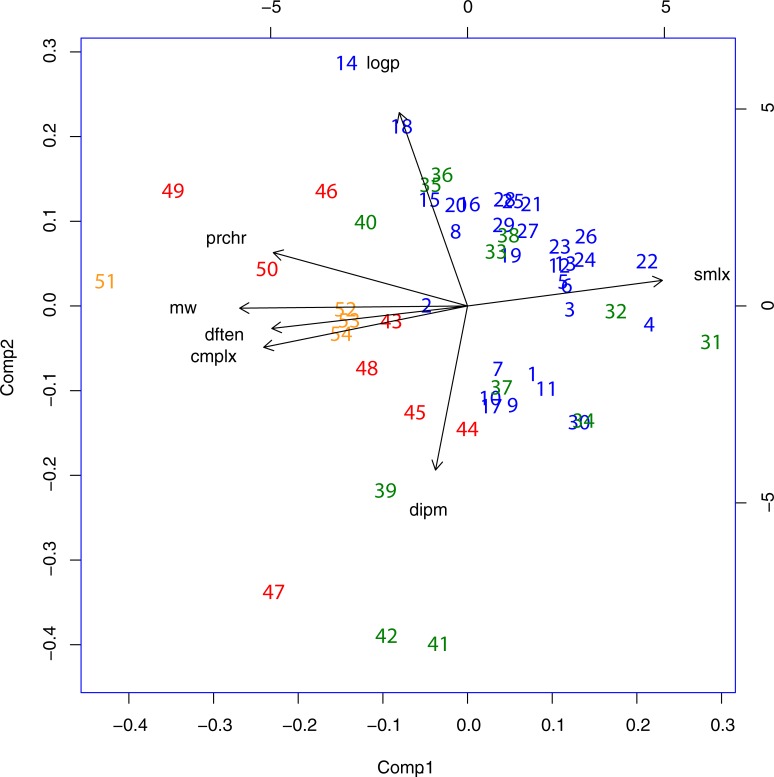
As in Fig 2 but with *mllr* and *mrchsn* left out in order to eliminate possible bias from these two sources.

Molecular complexity (*cmplx* in S1–S5 **Tables** and **Figs 2–4**) calculations based on Shannon entropy data using a previously published algorithm [24]were performed as described previously [7,8]. This method captures essential information concerning molecular structure including number and types of atoms, their connectivity, the number of multiple bonds and chirality. The issue of “types of atoms” needs some clarification: when a single heteroatomis replaced with a different heteroatom does not in itself change the complexity according to this scoring method. Example: selenocysteine has the same score as cysteine. But in reality the seleno- analogs are much more difficult to synthesise and they are electronically much more complex (see the above remarks about the quantum chemical calculations). They would for these reasons almost certainly “arrive later”.

In addition to this complexity metric, a much earlier methodology, parachor (*prchr* in S1–S5 **Tables** and **Figs 2–4**), which is based on contributions to surface tension from constituent molecular fragments, was revisited [25]and recruited for this study. This was because it was judged to be a way to enrich the statistical treatment, which in practice turned out to be the case (see below). As yet another supplement the above complexity calculations, Kolmogorov complexity data derived from SMILES descriptor strings were obtained using *bzip2* as a compression algorithm [26,27]. These scores are referred to as *smlx*. Lipophilicity (*logp*) was determined using the ALOGPS2.1 program [28].(http://www.vcclab.org/lab/alogps/).

There is a rich tradition of statistical studies of the properties of amino acids starting with the seminal work [29]that led to the development of the Sigma software for this purpose. In this work the R program (R Development Core Team, 2008) was used for all statistics calculations and plots (see **Figs 1–4** and S1–S5 **Tables**).

## Results

All data for all 58 compounds is shown in S1–S5 Tables. The ordering on this list is:

➢ Group 1 (blue / nos.: 1–30) non-standard amino acids and non-peptidogenic analogues found in either the Murchison (*mrchsn*) or Miller (*mllr*) sets,
➢ Group 2 (green / nos.: 31–42): members of the canonical set that were observed either on the Murchison meteorite or in the Miller experiment,
➢ Group 3 (red / nos.: 43–50) members of the canonical set that were not observed in these sets but which are extant,
➢ Group 4 (magenta / nos.: 51–54) amino acids not considered when the original genetic code was elaborated but which are now known to be present in many species in all three domains of life. These are pyrrolysine (standard abbreviation O, coded for by stop codon UAG). selenocysteine (standard abbreviation U, coded for by UGA, another stop codon) and selenomethionine (not coded for but produced enzymatically and herein abbreviated as J). In this set is included homoselenocysteine which is not found naturally (called herein homoU). No members of this group have been found on meteorites or in Miller-type experiments. They are all probably “post-RNA”, with the exception of homoU which has not been found/described.
➢ Group 5 (orange / nos.: 55–58) an entirely fictitious set of amino acids designed to study unexplored regions of “function space”.

Even a cursory examination of the data reveals that what characterises many members of Group 3 apart from the others is higher complexity scores (*cmplx*) compared to Group 2. This has been observed and commented on earlier [8]. It is notable that D (compound no. 41) and E (no. 42) are present in Group 2, but their amides N (no. 44) and Q (no. 45) respectively turn up first in Group 3. The complexity data do not explain this, neither do they explain why homocysteine (not encountered in proteins) is present in Group 1 while its simpler “canonical” sibling cysteine turns up first in Group 3. These observations suggest that there are other factors at work, as can be seen in the figures as discussed below. There is no immediate explanation as to why homocysteine (compound no 2) appears more prominently compared with its lower homologue cysteine (no. 43) but the answer may reside in the functional needs that dictate the choice of rotameric structure (discussed below). There may also be a stability/attrition aspect: cysteine may form in larger amounts but be more sensitive to degradation than homocysteine.

**Fig 1** shows the pairwise correlations between the various structural and quantum chemical variables, the statistical loadings (*cmplx*, *prchr*, *dipm*, *dften*, *logp*, *mllr*, *mrchsn*, *mw* and *smlx* defined as above). Good correlations can clearly be observed for *cmplx*, *prchr* with *mw* and mutually between *cmplx* and *prchr* even though these complexity scoring methods have a completely different origin. *C*omplexity score *smlx* seems rather to anticorrelate with *cmplx*, *prchr* and *mw*. This is because SMILES strings are already very compact consisting as they do of linear strings into which three dimensional chiral and heteroatomic structures are projected. The lengths and content of these strings do reflect molecular size and, of course, complexity and the compression algorithm conveys a measure of that complexity.

The variables *dipm* and *dften* derived from *ab initio* quantum chemical calculations as described above correlate differently with *mw* in particular: *dipm* is almost completely invariant with respect to *mw* while *dften* is more or less linearly dependent (molecular mass by its very nature implies greater intrinsic energy). (See **Fig 1**).

**Fig 2** shows the results of the first PCA analysis. What is observed here is a very clear clustering of the three groups. Group 3 (red color, nos. 43–50 in **S3 Table**) is the most distinct and is characterised by large negative values of Component 1. This component is dominated by *cmplx*, *mw*, *prchr* and *dften*. Along Component 2, the members of Group 3 straddle an intermediate range, especially avoiding being strongly lipophilic (*logp*). Generally, Group 2 (nos. 31–42, **S2 Table**) clusters at lower degrees of complexity (higher values of Component 1) and somewhat overlaps the Group 1 (blue) cluster. The latter is understandable given that Group 1 consists mainly of isomers or close analogs of the Class 2 amino acids. The two outliers, nos. 31 and 32 are the amino acids glycine and alanine respectively. They are the structurally simplest members of the group and can be regarded as the parents of all of the members of Groups 2, 3 and 4 and the “true” amino acids in Group 1. They were the most abundant in both *mllr* and *mrchsn*, and such simple compounds are likely to be found almost anywhere in interstellar space. Two other Group 2 outliers nos. 41 and 42 are aspartic and glutamic acids respectively, both highly polar, and they fulfil the needs of all subsequently formed proteins for providing an acidic function in their side chains. Also very important, especially for aspartate, the ability to bind metal ions so as to provide catalytic centers.

Group 2 and Group 3 together represent what has “survived”, the canonical set of amino acid types that we have today. But they are different, they seem each to reside on opposite sides of a “divide”. Group 2 mixes with Group 1 and these two sets are those that are found in interstellar space, on meteorites (*mchlsn*) or in laboratory experiments (*mllr*), whereas for Group 3, the factor that seems to be emerging as being most critical for their appearance seems to be complexity, in a general sense. More particularly: *cmplx*, *prchr* and *smlx* all serve to “stretch out” (**Fig 2**) the members of all three groups along an axis, Component 1, whose principle characteristic is clearly molecular complexity. This is the complexity that has to be “paid for” in order to fulfil the required functions. On the strength of these results there would be every reason to study these different measures of complexity in other contexts too. It may be that even the almost totally forgotten parachor still has a future.

Two further results are reported here. Firstly, the question of whether the coverage of functional space by the canonical set of 20 amino acid types is adequate for the task. Clearly it is, as judged *a posteriori*. Once these residues are incorporated into proteins there are additional functional requirements (catalysis in particular) that need to be met. This is accomplished by post-translational modifications. For the correct folding of the protein itself a number of residue types with lipophilic sidechains is required, both of aliphatic and aromatic type. But there is no need to go to extremes as in the fictitious compound 55 or the Miller (but not Murchison) compound 14. These extreme residue types are not used. Neither are the “alternatives” to the positively charged residues lysine/ariginine (compounds 56 and 57) or the negatively charged residues aspartate/glutamate (compound 58). The distributions of these non-standard “residue types” is shown in orange colour in **Fig 2**. But there are residue types not previously included in the “canonical set” that are used and they need to be considered. While there is no evidence that they played any kind of role in prebiotic chemical evolution or OoL itself, they certainly play a significant role today. These form the Group 4 and are included in the first PCA plot. All three appear as outliers colored magenta nos.: 51 (pyrrolysine) and 52–54 (seleno- compounds). The former owes its great complexity score to the presence of three chiral centers, while the seleno- compounds owe their positioning to the mere fact that selenium has replaced sulfur–the electronic energy (*dften*) and molecular weight (*mw*) are correspondingly higher.

The partitioning and clustering observed in Groups 1, 2 and 3 become clearer when the outliers pyrrolysine and the seleno- compounds are removed as shown in **Fig 3**.

Lastly a plot was made to see what the consequences of leaving *mllr* and *mrchsn* out, in order to meet any objections that they are somehow biasing the outcome. This plot is shown in **Fig 4**. There is some local mutual reshuffling of the points in the plot but it is not disrupted in any way. In fact whatever “bias” *mllr* and *mrchsn* may be introducing is necessary for this analysis because the former reflects experimental data and the latter is representative of real events from interstellar space.

In order to establish a mapping of the panel of compounds to chemical space a very comprehensive and diverse set of parameters was studied (*mw*, *cmplx*, *smlx*, *prchr*, *dften*, *dipm*, *logp*, *mllr* and *mrchsn*). Of these *cmplx* has been singled out as being important and is probably the main ingredient in the first principal component in the PCA plots of **Figs 2** and **3**. Complexity is an attribute of molecular structure but it is also an underlying cause of other attributes: ability to be synthesized at all [7]and to admit a wider variety of chemical functionalities. As such, it can also be seen as a cost factor. In order to elucidate this point, a new plot was made whereby *cmplx* was omitted as an individual parameter but instead, the remaining parameters were each divided by their corresponding value for *cmplx*. This produces a normalized version of the previous two plots, which allows greater emphasis on the effect of the remaining attributes.

As is shown in **Fig 4** Group 1 and 2 mutually overlap very strongly as do Group 3 and 4, but now there is a very clear separation between these two constellations (the “divide” referred to above). This suggests that there is a real partition between subsets of compounds known to turn up in outer space and those only known in a terrestrial environment. This does not mean that they necessarily are products of biosynthesis–quite the converse, as discussed below. But it does support the notion that there are certain physical features of planet Earth (and possibly certain exoplanets) that are conducive to the production of these more complex compounds. Some new features of the plot in **Fig 4** require comment and explanation: Compound 43 (cysteine) now appears in the cluster formed by the “simple” groups 1 and 2. This is in accord with the fact that it has a complexity about the same as serine (and its “synthesizability score” is even lower than that of serine [7]). Compounds 41 and 42, which belong to Group 2 have now migrated into the Group 3 + 4 cluster. The amino acids, aspartic acid and asparagine were assigned to Group 2 on the strength of their strong presence in *mrchsn* and *mllr* but their complexity and synthesizability scores put them at the borderline between Groups 2 and 3.

To summarize, **Figs 2–4** show how the amino acids (Group 2 (green) compounds 31–42 and Group 3 (red) 43–50) are distributed relative to other prebiotic compounds (Group 1 (blue) 1–30). The multivariate analysis has identified two principal orthogonal variables, component 1 which clearly expresses complexity (as defined collectively by the variables *cmplx*, *prchr*, *smlx*, *mldx*, also *prchr and dftn* while component 2 equally clearly has the character of hydrophobicity *contra* polarity (earlier proposed by Higgs and Pudritz, 2009) as demonstrated by the direction of the *logp* and *dipm* vectors. Group 2 has a disjoint distribution, with A (32) and G (31) located separately from the main group, as already mentioned. They are small (low complexity) and almost totally devoid of hydrophobic character (*logp*). The other outlier set, D (41) and E (42) are extremely polar in character. Otherwise, Group 2 occupies the centre stage overlapping with Group 1 to some degree, but mostly distributed towards higher complexity compared with Group 1. The functionally simplest peptide structures can form spontaneously from amino acids in this group (especially transmembrane peptides [7]). Later, when the more complex Group 3 amino acid types appear, they too can be incorporated into peptides and ultimately into proteins. They are spread over the hydrophobicity/polarity (component 2) spectrum, as required, and their greater complexity reflects the demands for ever greater functional exigencies.

A final remark that belongs to the **Results** section is to note the overlaps between serine no. 34 and its isomer no. 30, leucine no. 36 and its homolog nor leucine no. 20 and valine no. 33 and its isomers no. 19 and 29. Close proximity is also noticeable between cysteine no. 43 and its homolog homocysteine no. 2 and similarly between serine no. 34 and its homolog homocysteine no. 1. There is no immediate explanation on complexity grounds (for example): homocysteine was found in *mllr* while the synthetically simpler cysteine was not. This has been discussed above. One reason for that may have to do with available/required rotamer structure (discussed below) or alternatively stability and/or rate of attrition. The situation in relation to the analogous selenium compounds may well be similar.

## Discussion

The claim here is that the present analysis serves as an explanation as to why the set of 20 now naturally occurring amino acid residue types, the canonical set, has its particular membership. In one or another way there seems to be a need to incorporate sufficient complexity (concomitant with, and indicative of, the need to retain important chemical functionalities) while still needing to remain within the confines of a stable and synthetically accessible structural framework. Complexity almost certainly correlates strongly with molecular recognition propensities, critical for protein folding and function. Only a very few highly polar residues (*dipm*) are needed, aspartate and glutamate, referred to above, and no. 47 arginine. There is no need for any extra charged residues of the type deliberately constructed by the author to test the limits of what is relevant and needed (nos. 56–58 or similar). There is a tendency to avoid too high lipophilicity (*logp*), which anyway would cause the solubility and redundancy problems alluded to above. Aromaticity is already adequately catered by phenylalanine (no need e.g. for no. 55 or its like). Every effort has been made to explore an extended repertoire of “amino acid structural space” but no novel or useful functionalities have been discovered. Within the canonical set there is a sufficient balance between the two extremes of polarity and of lipophilicity (/aromaticity). There is more than a hint of a “Goldilocks principle” at work here. The notion of parsimony in terms of not requiring the use of a greater population of building blocks then is strictly necessary may well apply here, as it does throughout all of nature.

There are two major subsets of amino acid residue types, however, Group 2 (the *mrchsn*/*mllr* set of 12) and Group 3 (the remaining 8 that turned up later in OoL) that are different in character. For Group 3 complexity seems to be the dominating factor and this complexity may simply be too great to have allowed their production under the harsh conditions of space or in laboratory glassware under extreme conditions. The notion has been advanced and convincingly discussed in the context of OoL, that this latter group of residue types needed special conditions involving water, tides, catalysis and cycles of pyrolysis in order to be produced (Bywater, 2012). This separation between Group 3 and Groups 1 & 2 (the “divide” referred to above) brings to mind another interfacial region discussed in that paper: the notion that beaches provided the substratum for these events to occur [30]. Mention should also be made of other suggestions including undersea hydrothermal vents, or delivery from outer space [6]also discussed in [30].

One may legitimately ask the question: why just 20? This question has indeed already been posed earlier [16]. The standard apparatus for living systems, based on DNA caters for 64 possible amino acid types. But of course, any “language” needs more than just the constituent “letters”; punctuation is important too. Stop and start codons, in the parlance of DNA. So, let us say that, of the 64 codons that DNA provides us with, 61 “codon products” have the task of catering for all of the structural and functional needs of the translated and fully-fledged proteins. But the protein world has not taken up the offer of having 61 alternatives. It was deemed (very early on) that 20 is enough. Why? One clue to solving this conundrum is that: for every molecule with a given structure, and equipped with a certain set of chemical functionalities, there is a sense in which energetically well-separated conformers of the any given molecule, especially in the environment of a well packed protein structure, can be regarded as separate, individual, molecular species. At least for the purposes of function. Although there are entire databases of experimental side-chain rotamer data that rather “smear out” this data [31,32](there are crystal- and core-packing reasons for this as well as experimental errors) the side-chains of the constituent amino acid residues do exhibit such a set of “energetically well-separated conformers”. These are based on the classical rotamer states [33]of any aliphatic chain, the *gauche+*, *gauche-* or *trans* arrangements: http://www.cryst.bbk.ac.uk/PPS95/course/9_quaternary/3_geometry/conform.html

This kind of model has been used to construct theoretical rotamer libraries in the past. The first of these [34]arrived at the following conclusions: “17 of the 20 amino acids (omitting Met, Lys and Arg) can be represented adequately by 67 side-chain rotamers” and in another study [35], the number of rotamer states was shown by a dynamic cluster analysis of a large database of known crystallographic structures to be ~109, but if one discards states with less than 10% occupancy the number comes down to 59. Even the “smoothed out” database [32]displays a distinct pattern with 53 minima in all, as shown in the histogram data for the χ-1 torsion angle. So we seem to be bracketing this “desired number” of 61. If it is a desired number. It means that there is some redundancy in the translation code, which we are well aware of. The need to populate different energetically mutually accessible, but effectively distinct, rotameric states for functional and packing reasons and the ability of members of the canonical set to do so could have eliminated the need for there to be more than 20 residue types. It must be made clear here that while a given amino acid type, or any chemical compound for that matter, has a unique identity, at the level of function the compound can have many identities with very different electronic, steric, recognition/affinity and certainly biological properties depending on rotameric states, especially if these are frozen or restricted as for the case of amino acids in proteins. This may incidentally be the explanation for the selections referred to at the end of **Results**: serine rather than homoserine, cysteine before homocysteine, valine and leucine rather than their isomers. These alternative molecular arrangements simply did not offer the required functional repertoire. Function is after all the deciding factor in evolution.

Further, there may have been more than the “canonical 20” amino acid types that emerged as the leading contenders (for making functional peptides and proteins) these “supernumary” amino acid types may have been used at some time to fulfil the role defined by some rotamer or other and may therefore have had individual codons. But if it then turned out that the function could be performed satisfactorily by another (simpler) amino acid in a suitable rotameric state then “supernumary” could have become “superfluous”. We have no record of this, and anyway, it is entirely feasible that such a culling out of expendable structures may equally well have occurred prebiotically, and probably did in view of the protocell model proposed previously [7,8]. So there is, once again, no necessity to invoke biochemical mechanisms (proteins, RNA) in this process.

The explanation for codon redundancy as presented here is novel but the need to provide some/any explanation is by no means new. According to earlier arguments on this subject there could be any number of other reasons for needing, or just opportunistically using, this redundancy. These include the question of whether the local protein folding process is affected by synonymous codon usage (discussed in detail elsewhere [27]), suggestions of the type “the genetic code is constructed to limit errors in protein production” or it has to do with items like the translational pausing, the rate of growth of microorganisms or numerous other explanations based on evolution theory. The notion of there being some kind of “second message” encoded in the DNA sequence has been advanced many times but no consensus has ever been established. Whenever such alternatives are up for debate it is helpful to invoke the well-established Ockham principle: the simplest explanation should in the first instance be preferred over more elaborate ones. All 20 amino acid types were in existence prior to the advent of RNA and were in use in the synthesis of oligopeptides [36]where there would be use for different rotamer states. The simplest explanation would therefore seem to be that the different rotamers states were already exercising different functions prior to th advent of codons. The work undertaken here suggests that, whatever choice was made, it was determined by the functional advantages that were thereby made available and the final decision was dictated according to the standard evolutionary rules of elimination/survival first at a chemical level, later in a biological setting.

Before concluding, some remarks should be made about why the question of prebiotic chemical evolution is so important. Taking the entire OoL process into account, from ~4.2 Gyrs ago onwards we can obviously not discard the “RNA world” (commencing ~3.9 Gyrs ago), but we definitely need to consider what went before it [8]. A recent review [37]tracks the progress” of evolution and post-evolution of the genetic information system through emergence of life. The major stages traversed include prebiotic synthesis, functional RNA selection by metabolite, RNA world, peptidated RNA world, co-evolution of genetic code and amino acid biosynthesis, last universal common ancestor, Darwinian evolution and synthetic life”. As such, the review makes a sterling effort to cover OoL history and the coverage is excellent as far as the RNA world period is concerned. But it is all emphatically "post-RNA" as declared in the title of the paper itself ("Emergence of life: from functional RNA selection to natural selection and beyond"). For the present discussion, which largely focuses on the prebiotic world, the RNA world as described is irrelevant. The author [37]admits this inadvertantly by referring to work [38]that emphasized the inadequacy of the RNA world. Instead a Coevolution Theory is proposed which suggests that of the 20 canonical amino acids in the protein alphabet, G, A, S, D, E, V, L, I, P and T represent Phase 1 amino acids that were early-arrivals supplied by the environment, and the remaining ten, F, Y, R, H, W, N, Q, K, C and M, represent late-arrival, biosynthesis-derived, Phase 2 amino acids. It is interesting that the set of amino acid types is very similar to that found earlier by the present author (Bywater, 2012) based on complexity calculations. There was a "set 1" consisting of G, A, V, S, I, L, C, T, P, K, M and "set 2" N, Q, D, E, F, R, H, Y, W. But there are differences, and these differences are significant. In the latter scheme N, Q, D and E are in the second set—this assignment is made on the grounds of molecular complexity (formation of double bonds is “expensive”). Note that the latter scheme envisages only chemical evolution—prebiotic synthesis in the total absence of enzymes or ribozymes. In contrast to this, the supposed "early arrival" (Phase 1) is defined as prebiotic while late arrival (Phase 2) is said to require biosynthesis (Wong, 2014). But there are no grounds for such an assumption. For example, formation of cysteine is said to require an enzyme (a complex post-RNA construct, which would surely require at least some of the ("Phase 2") aromatic residue types for stability and function) but its close relative serine does not, evidently. The paper abounds with original coinages not used before or elsewhere such as inventive biosynthesis and pretrans synthesis requiring catalysis by either ribozyme or enzyme.

It should be recognized that RNA is a relatively late development and enzymes certainly are. The latter are built up partly from the very amino acid types that are said to require enzymes for their creation. But there is no need to invoke these mechanisms in order to explain how prebiotic chemical evolution took place. It is simply that the second set took longer to be produced and they probably needed the assistance of terrestrial catalysts. There is an abundance of catalysts of the kind needed to promote these synthetic processes in the mineral world, for example on beaches [30]. Repeated cycles of chemical synthesis were followed by sorting and selection mechanisms. Natural selection is not restricted to the (post-)RNA world, natural selection by chemical evolution was a necessary and evidently sufficient precondition to OoL itself.

A further recent review that discusses coverage of chemical space state [9] that “Sets that cover chemistry space better than the genetically encoded alphabet are extremely rare and energetically costly”. This accords very well with the conclusions from the present study. Further, these authors stated “The amino acids used for constructing coded proteins may represent a largely global optimum, such that any aqueous biochemistry would use a very similar set.” In this work I have been able to distinguish the two subsets of the amino acids into a primitive subset that could easily be made anywhere in space and a more complex subset that may have existed elsewhere (but have never been found) but very likely could have been produced by one or other of the aforementioned geological processes, undersea vents and tidal beaches (both available on Earth and by inference on sufficiently Earth-like exoplanets). Although the second subset is characterized by greater complexity such that they mostly likely require some sort of catalysis to speed up their production, there is no need to assume that the catalysts were enzymes or ribozymes. Mineral catalysts would be adequate to do the job. So “any aqueous biochemistry would use a very similar set” can be replaced with “any aqueous chemistry would produce a very similar set” and it could all take place prebiotically. There is no need to assume that RNA and or enzymes were involved and indeed, enzymes by demand would need to contain amino acid types such as H, Y, W and others in the second subset.

To summarize the foregoing: any proposal that the selection mechanism was based on metabolic evolution [9]or pretrans synthesis [37]amounts to a circular argument–the metabolic evolution would have had to take place after these amino acid types had been generated. I have instead proposed a prebiotic chemical evolution. Selection after multiple (myriads) cycles of synthesis, degradation, separation and new synthesis [7].

It would have been possible to include many more amino acids and amino acid like compounds and indeed this has been done by others and also in this work, but these results are not reported. The PCA plots are already very crowded and no advantage was to be gained by simply increasing the redundancy. Furthermore, as explained above, chemical evolution required strict parsimony and avoidance of the elaboration of unnecessary complexity through chain-branching, extra chiral centers and the like. Instead the focus has been on the types of compounds that are known to be important. The four hypothetical compounds (nos. 55–58) were included in order to explore the outer envelope of the chemical space under discussion and indeed that is where these compounds reside, but not much more would be gained by further “engineering” of this sort. In the work mentioned above [9]a much larger compound library comprising 1913 compounds was studied. These authors used only three descriptors (size, hydrophobicity, and charge.) to partition this large set. It is not clear how this portioning was distributed in chemical space except for the statement about “aqueous biochemistry” discussed above which suggested that there was a considerable degree of convergence onto what became the canonical 20. Of these 20 amino acid types, 17 possess side chains that can exist in one or more different rotameric states. In proteins where there is a degree of solid-state character (as opposed to liquid or gas phase) these rotameric states are to be regarded as separate individual molecular species especially in regard to recognition properties and biological function. To the extent that the overall number of accessible rotameric states for these 17 amino acids species could well be ~61 is an expression of the need for the functional repertoire required by successful living organisms. So, well in advance of the emergence of issues such as code degeneracy, the amino acids were stereochemically predisposed by these rotamer states to fill the required function space (having cardinality ~61) using only the canonical 20. In passing, this solves the conundrum as to why it was necessary (much later) for there to be a triplet code as in DNA. A duplex code would provide for only 16 distinct chemical species, far short of ~61 and also short of the 20 that were already available and were later utilised and needed by very primitive living systems.

## Conclusions

The key point that emerges from the work reported herein is that there exists a set of fundamental physicochemical imperatives that dictated, prebiotically, the choice of the canonical set of 20 amino acid residue types. The guiding principle throughout the process of selection of the chemical compounds is assumed to be energy-related and above all based on parsimony whereby the simplest possible structures that have value in terms of function are retained. Redudancy and unnecessary complexity are eschewed. This is what underlies the major postulate of this work as expressed in the title. The claim is based on a penetrating quantum chemical and chemoinformatic sudy of a large panel of candidate substances followed by statistical analysis of complexity and property scores for these substances which identifies the most likely chemicals that appeared prebiotically, most prominently the 20 amino acid types that are still with us. If it were possible for life to be sustained on a platform consisting of only 16 amino acid types, which ones could be discarded?

## Supporting information

S1 Table Group 1: (nos.: 1–30) non-standard amino acids and non-peptidogenic analogues found in either the Murchison (*mrchsn*) or Miller (*mllr*) sets. (DOC)

S2 Table Group 2: (nos.: 31–42) members of the canonical set that were observed either on the Murchison meteorite or in the Miller experiment. (DOC)

S3 Table Group 3: (nos.: 43–50) members of the canonical set that were not observed in these sets but which are extant. (DOC)

S4 Table Group 4: (nos.: 51–54) amino acids not considered when the original genetic code was elaborated but which are now known to be present in many species in all three domains of life. These are pyrrolysine (standard abbreviation O, coded for by stop codon UAG). selenocysteine (standard abbreviation U, coded for by UGA, another stop codon) and selenomethionine (not coded for but produced enzymatically and herein abbreviated as J). In this set is included homoselenocysteine which is not found naturally (called herein homoU). No members of this group have been found on meteorites or in Miller-type experiment.
(DOC)

S5 Table Group 5: (nos.: 55–58) an entirely fictitious set of amino acids designed to study unexplored regions of “function space. (DOC)

S6 Table Chemical names for compounds in S1–S5 Tables. (DOC)
