# Supplementary material for: Why twenty amino acid residue types suffice(d) to support all living systems
Source: PLoS One. 2018 Oct 15;13(10):e0204883. doi: 10.1371/journal.pone.0204883 (PMC6188899; doi:10.1371/journal.pone.0204883)
Supplement: S1 Table — (DOC) [file pone.0204883.s001.doc]

| cpd | num | mw | cmplx | smlx | prchr | dften | dipm | logp | mllr | mrchsn |
| --- | --- | --- | --- | --- | --- | --- | --- | --- | --- | --- |
| homoS | 1 | 119 | 70 | 2.70 | 398 | 438.45 | 12.49 | -3.04 | 0.50 | 0.00 |
| homoC | 2 | 135 | 73 | 2.70 | 435 | 761.38 | 12.09 | -2.07 | 0.50 | 0.00 |
| gABA | 3 | 103 | 49 | 2.70 | 379 | 363.19 | 26.64 | -1.91 | 0.50 | 7.00 |
| bA | 4 | 89 | 43 | 3.17 | 308 | 323.87 | 22.11 | -2.28 | 4.30 | 10.00 |
| bAnBA | 5 | 103 | 50 | 2.58 | 383 | 363.20 | 16.23 | -1.95 | 0.10 | 5.00 |
| bAiBA | 6 | 103 | 50 | 2.63 | 383 | 363.19 | 16.18 | -2.01 | 0.50 | 7.00 |
| alloT | 7 | 119 | 71 | 2.25 | 403 | 438.45 | 16.30 | -2.72 | 0.20 | 0.00 |
| alloI | 8 | 131 | 56 | 2.10 | 468 | 441.84 | 15.28 | -1.37 | 1.20 | 0.00 |
| aHgABA | 9 | 119 | 70 | 2.48 | 398 | 438.44 | 27.74 | -2.77 | 17.00 | 0.00 |
| agDABA | 10 | 119 | 64 | 2.17 | 444 | 419.01 | 27.07 | -2.78 | 7.60 | 0.00 |
| abDAPA | 11 | 105 | 59 | 2.29 | 374 | 379.69 | 14.92 | -3.05 | 1.50 | 0.00 |
| aAnBA | 12 | 103 | 50 | 2.54 | 383 | 363.20 | 15.44 | -1.78 | 61.00 | 19.00 |
| aAiBA | 13 | 103 | 51 | 2.70 | 399 | 363.19 | 15.17 | -1.85 | 7.00 | 33.00 |
| aAhept | 14 | 159 | 71 | 2.29 | 667 | 520.49 | 15.36 | 0.17 | 0.30 | 0.00 |
| tBuL | 15 | 131 | 69 | 2.13 | 536 | 441.84 | 14.93 | -1.13 | 0.01 | 0.00 |
| pipec | 16 | 129 | 76 | 2.58 | 479 | 440.64 | 16.09 | -1.10 | 0.01 | 0.00 |
| O | 17 | 132 | 59 | 2.54 | 500 | 457.89 | 15.65 | -3.52 | 0.01 | 0.00 |
| NmeG | 18 | 145 | 63 | 2.33 | 596 | 481.16 | 15.70 | -0.46 | 0.00 | 0.00 |
| norV | 19 | 117 | 56 | 2.48 | 454 | 402.52 | 15.52 | -1.75 | 14.00 | 14.00 |
| norL | 20 | 131 | 63 | 2.42 | 525 | 441.84 | 15.65 | -1.25 | 1.40 | 0.00 |
| NnprG | 21 | 117 | 56 | 2.90 | 453 | 402.52 | 16.67 | -1.24 | 0.50 | 0.00 |
| NmeG | 22 | 89 | 40 | 3.17 | 312 | 323.87 | 16.03 | -1.81 | 12.50 | 7.00 |
| NmeA | 23 | 103 | 55 | 2.54 | 351 | 363.16 | 16.40 | -1.43 | 3.40 | 3.00 |
| NmebA | 24 | 103 | 50 | 3.00 | 383 | 363.19 | 22.95 | -1.57 | 1.00 | 0.00 |
| NiprG | 25 | 117 | 56 | 2.64 | 458 | 402.52 | 16.25 | -1.16 | 0.50 | 0.00 |
| NetG | 26 | 103 | 50 | 3.00 | 383 | 363.19 | 16.34 | -1.55 | 6.80 | 6.00 |
| NetbA | 27 | 117 | 56 | 2.85 | 453 | 402.52 | 23.19 | -1.29 | 0.05 | 0.00 |
| NetA | 28 | 117 | 57 | 2.52 | 458 | 402.52 | 16.01 | -1.11 | 0.05 | 0.00 |
| isoV | 29 | 117 | 57 | 2.46 | 460 | 402.52 | 15.15 | -1.42 | 1.00 | 11.00 |
| isoS | 30 | 105 | 63 | 2.73 | 327 | 399.12 | 22.32 | -3.14 | 1.20 | 0.00 |

**S1 Table**

Group 1: Non-standard amino acids and non-peptidogenic analogues found in either the

Murchison (*mrchsn*) or Miller (*mllr*) sets.
