# Supplementary material for: Why twenty amino acid residue types suffice(d) to support all living systems
Source: PLoS One. 2018 Oct 15;13(10):e0204883. doi: 10.1371/journal.pone.0204883 (PMC6188899; doi:10.1371/journal.pone.0204883)
Supplement: S2 Table — (DOC) [file pone.0204883.s002.doc]

| cpd | num | mw | cmplx | smlx | prchr | dften | dipm | logp | mllr | mrchsn |
| --- | --- | --- | --- | --- | --- | --- | --- | --- | --- | --- |
| G | 31 | 75 | 36 | 3.35 | 237 | 284.55 | 16.07 | -2.66 | 100.00 | 100.00 |
| A | 32 | 89 | 43 | 2.65 | 313 | 323.87 | 15.70 | -2.23 | 180.00 | 36.00 |
| V | 33 | 117 | 57 | 2.30 | 459 | 402.52 | 15.28 | -1.67 | 4.40 | 0.00 |
| S | 34 | 105 | 63 | 2.73 | 327 | 399.13 | 14.23 | -3.42 | 1.10 | 0.00 |
| I | 35 | 131 | 64 | 2.10 | 530 | 441.84 | 14.97 | -0.96 | 1.10 | 0.00 |
| L | 36 | 131 | 64 | 2.29 | 530 | 441.84 | 15.54 | -0.88 | 2.60 | 0.00 |
| T | 37 | 119 | 71 | 2.25 | 403 | 438.46 | 13.91 | -3.02 | 0.20 | 0.00 |
| P | 38 | 115 | 72 | 2.48 | 408 | 401.31 | 16.18 | -1.31 | 0.30 | 22.00 |
| K | 39 | 146 | 77 | 2.24 | 571 | 497.66 | 89.00 | -1.73 | 0.01 | 0.00 |
| M | 40 | 149 | 81 | 2.54 | 486 | 800.70 | 11.88 | -1.13 | 0.10 | 0.00 |
| D | 41 | 133 | 103 | 2.21 | 372 | 512.07 | 86.42 | -2.95 | 7.70 | 13.00 |
| E | 42 | 147 | 110 | 2.13 | 443 | 551.39 | 88.92 | -2.84 | 1.70 | 20.00 |

**S2 Table**

Group 2: members of the canonical set that were observed either on the Murchison meteorite or in the Miller experiment.
