# Supplementary material for: Why twenty amino acid residue types suffice(d) to support all living systems
Source: PLoS One. 2018 Oct 15;13(10):e0204883. doi: 10.1371/journal.pone.0204883 (PMC6188899; doi:10.1371/journal.pone.0204883)
Supplement: S3 Table — (DOC) [file pone.0204883.s003.doc]

| cpd | num | mw | cmplx | smlx | prchr | dften | dipm | logp | mllr | mrchsn |
| --- | --- | --- | --- | --- | --- | --- | --- | --- | --- | --- |
| C | 43 | 121 | 68 | 1.51 | 364 | 722.05 | 13.15 | -1.76 | 0.00 | 0.00 |
| N | 44 | 132 | 96 | 2.33 | 406 | 492.65 | 13.08 | -3.38 | 0.00 | 0.00 |
| Q | 45 | 146 | 103 | 2.29 | 477 | 531.97 | 18.71 | -3.07 | 0.00 | 0.00 |
| F | 46 | 165 | 125 | 2.16 | 547 | 554.98 | 14.28 | -0.71 | 0.00 | 0.00 |
| R | 47 | 174 | 125 | 2.10 | 684 | 607.23 | 88.08 | -2.81 | 0.00 | 0.00 |
| H | 48 | 155 | 129 | 2.06 | 473 | 548.96 | 12.40 | -2.58 | 0.00 | 0.00 |
| W | 49 | 204 | 190 | 1.90 | 636 | 686.59 | 11.12 | -0.52 | 0.00 | 0.00 |
| Y | 50 | 181 | 154 | 2.00 | 559 | 630.24 | 15.07 | -1.34 | 0.00 | 0.00 |

**S3 Table**

Group 3: members of the canonical set that were not observed in these sets but which are extant,
