# Supplementary material for: Why twenty amino acid residue types suffice(d) to support all living systems
Source: PLoS One. 2018 Oct 15;13(10):e0204883. doi: 10.1371/journal.pone.0204883 (PMC6188899; doi:10.1371/journal.pone.0204883)
Supplement: S4 Table — These are pyrrolysine (standard abbreviation O, coded for by stop codon UAG). selenocysteine (standard abbreviation U, coded for by UGA, another stop codon) and selenomethionine (not coded for but produced enzymatically and herein abbreviated as J). In this set is included homoselenocysteine which is not found naturally (called herein homoU). No members of this group have been found on meteorites or in Miller-type experiment. (DOC) [file pone.0204883.s004.doc]

| cpd | num | cmplx | prchr | dipm | dften | logp | mllr | mrchsn | mw | smlx |
| --- | --- | --- | --- | --- | --- | --- | --- | --- | --- | --- |
| O | 51 | 263 | 1019 | 23.71 | 860.45 | 0.09 | 0.00 | 0.00 | 255 | 0.68 |
| U | 52 | 68 | 377 | 11.53 | 2753.85 | -2.26 | 0.00 | 0.00 | 168 | 0.46 |
| J | 53 | 81 | 499 | 9.49 | 2832.55 | -1.74 | 0.00 | 0.00 | 196 | 0.43 |
| homoU | 54 | 73 | 448 | 10.57 | 2793.13 | -2.04 | 0.00 | 0.00 | 182 | 0.50 |

**S4 Table**

Group 4. Amino acids not considered when the original genetic code was elaborated but which are now known to be present in many species in all three domains of life. These are pyrolysine ( standard abbreviation O, coded for by stop codon UAG). selenocysteine (standard abbreviation U, coded for by UGA, another stop codon) and selenomethionine (not coded for but produced enzymatically and herein abbreviated as J). In this set is included homoselenocysteine which is not found naturally (called herein homoU). No members of this group have been found on meteorites or in Miller-type experiments.
