# Supplementary material for: Why twenty amino acid residue types suffice(d) to support all living systems
Source: PLoS One. 2018 Oct 15;13(10):e0204883. doi: 10.1371/journal.pone.0204883 (PMC6188899; doi:10.1371/journal.pone.0204883)
Supplement: S5 Table — (DOC) [file pone.0204883.s005.doc]

| cpd | num | mw | cmplx | smlx | prchr | dften | dipm | logp | mllr | mrchsn |
| --- | --- | --- | --- | --- | --- | --- | --- | --- | --- | --- |
| NPTH | 55 | 216 | 197 | 1.35 | 720 | 708.55 | 10.69 | -1.55 | 0.00 | 0.00 |
| Ht2 | 56 | 156 | 140 | 1.95 | 454 | 564.90 | 10.64 | -1.83 | 0.00 | 0.00 |
| Ht5 | 57 | 156 | 140 | 1.98 | 454 | 564.90 | 9.70 | -2.00 | 0.00 | 0.00 |
| Hte | 58 | 157 | 149 | 1.93 | 430 | 580.92 | 10.569 | -1.99 | 0.00 | 0.00 |

**S5 Table**

Group 5. An entirely fictitious set of amino acids designed to study unexplored regions of “function space”.
