# Supplementary material for: Why twenty amino acid residue types suffice(d) to support all living systems
Source: PLoS One. 2018 Oct 15;13(10):e0204883. doi: 10.1371/journal.pone.0204883 (PMC6188899; doi:10.1371/journal.pone.0204883)
Supplement: S6 Table — (DOC) [file pone.0204883.s006.doc]

| cpd | num | Chemical names for compounds in  S1 – S5 Tables |
| --- | --- | --- |
| homoS | 1 | L-homoserine |
| homoC | 2 | L-homocysteine |
| gABA | 3 | γ-aminobutyric acid |
| bA | 4 | β-alanine |
| bAnBA | 5 | β-Amino-n-butyric acid |
| bAiBA | 6 | β-Aminoisobutyric acid |
| alloT | 7 | L-allothreonine |
| alloI | 8 | L-alloisoleucine |
| aHgABA | 9 | α-hydroxy-γ-aminobutyric acid |
| agDABA | 10 | α,γ-diaminopropionic acid |
| abDAPA | 11 | α,β-diaminopropionic acid |
| aAnBA | 12 | β-Amino-n-butyric acid |
| aAiBA | 13 | α-Aminoisobutyric acid |
| aAhept | 14 | α-Amino-n-heptanoic acid |
| tBuL | 15 | t-leucine |
| pipec | 16 | pipecolic acid |
| O | 17 | L-ornithine |
| NmeG | 18 | sarcosine |
| norV | 19 | L-norvaline |
| norL | 20 | L-norleucine |
| NnprG | 21 | N-propyl glycine |
| NmeG | 22 | N-methyl glycine |
| NmeA | 23 | N-methyl alanine |
| NmebA | 24 | N-methyl β-alanine |
| NiprG | 25 | N-isopropyl glycine |
| NetG | 26 | N-ethyl glycine |
| NetbA | 27 | N-ethyl-β-alanine |
| NetA | 28 | N-ethylalanine |
| isoV | 29 | L-Isovaline |
| isoS | 30 | L-Isoserine |
| G | 31 | glycine |
| A | 32 | L-alanine |
| V | 33 | L-valine |
| S | 34 | L-serine |
| I | 35 | L-isoleucine |
| L | 36 | L-leucine |
| T | 37 | L-threonine |
| P | 38 | L-proline |
| K | 39 | L-lysine |
| M | 40 | L-methionine |
| D | 41 | L-aspartic acid |
| E | 42 | L-glutamic acid |
| C | 43 | L-cysteine |
| N | 44 | L-asparagine |
| Q | 45 | L-glutamine |
| F | 46 | L-phenylalanine |
| R | 47 | L-arginine |
| H | 48 | L-histidine |
| W | 49 | L-tryptophan |
| Y | 50 | L-tyrosine |
| O | 51 | L-pyrrolysine |
| U | 52 | L-selenocysteine |
| J | 53 | L-selenomethionine |
| homoU | 54 | L-homoselenocysteine |
| NPTH | 55 | 2-amino-3-naphthyl-propionic acid |
| Ht2 | 56 | 2-Amino-3-(1H-1,2,3,triazol-4-yl)propanoic acid |
| Ht5 | 57 | 2-Amino-3-(1H-1,3,5,triazol-4-yl)propanoic acid |
| Hte | 58 | 1H-1,2,3,5,tetrazol-4-yl)propanoic acid |

**S6 Table.** Chemical names for compounds in **S1 – S5 Tables**.
